# Impact of the COVID-19 Pandemic on Cancer Care: A Global Collaborative Study

**DOI:** 10.1200/GO.20.00351

**Published:** 2020-09-28

**Authors:** Abdul Rahman Jazieh, Hakan Akbulut, Giuseppe Curigliano, Alvaro Rogado, Abdullah Ali Alsharm, Evangelia D. Razis, Layth Mula-Hussain, Hassan Errihani, Adnan Khattak, Roselle B. De Guzman, Clarissa Mathias, Mohammad Omar Farouq Alkaiyat, Hoda Jradi, Christian Rolfo, on behalf of the International Research Network on COVID-19 Impact on Cancer Care

**Affiliations:** ^1^ Department of Oncology, King Saud bin Abdulaziz University for Health Sciences and King Abdullah International Medical Research Center, Riyadh, Kingdom of Saudi Arabia; ^2^ Ankara University School of Medicine, Department of Medical Oncology, Ankara, Turkey; ^3^ European Institute of Oncology, Istituto di Ricovero e Cura a Carattere Scientifico, and University of Milan, Milan, Italy; ^4^ Fundación ECO, Madrid, Spain; ^5^ King Fahad Medical City, Riyadh, Kingdom of Saudi Arabia; ^6^ Hygeia Hospital, Athens, Greece; ^7^ Radiation Oncology, Faculty of Medicine, University of Ottawa, Ottawa, Ontario, Canada; ^8^ National Institute of Oncology, Mohammed V University, Rabat, Morocco; ^9^ Fiona Stanley Hospital, Perth, Western Australia, Australia; ^10^ Manila Central University-Filemon D. Tanchoco Medical Foundation Hospital, Caloocan City, Philippines; ^11^ Núcleo de Oncologia da Bahia, Grupo Oncoclínicas, Salvador, Bahia, Brazil; ^12^ King Saud Bin Abdulaziz University for Health Sciences, Riyadh, Kingdom of Saudi Arabia; ^13^ University of Maryland School of Medicine, Baltimore, MD

## Abstract

**PURPOSE:**

The COVID-19 pandemic affected health care systems globally and resulted in the interruption of usual care in many health care facilities, exposing vulnerable patients with cancer to significant risks. Our study aimed to evaluate the impact of this pandemic on cancer care worldwide.

**METHODS:**

We conducted a cross-sectional study using a validated web-based questionnaire of 51 items. The questionnaire obtained information on the capacity and services offered at these centers, magnitude of disruption of care, reasons for disruption, challenges faced, interventions implemented, and the estimation of patient harm during the pandemic.

**RESULTS:**

A total of 356 centers from 54 countries across six continents participated between April 21 and May 8, 2020. These centers serve 716,979 new patients with cancer a year. Most of them (88.2%) reported facing challenges in delivering care during the pandemic. Although 55.34% reduced services as part of a preemptive strategy, other common reasons included an overwhelmed system (19.94%), lack of personal protective equipment (19.10%), staff shortage (17.98%), and restricted access to medications (9.83%). Missing at least one cycle of therapy by > 10% of patients was reported in 46.31% of the centers. Participants reported patient exposure to harm from interruption of cancer-specific care (36.52%) and noncancer-related care (39.04%), with some centers estimating that up to 80% of their patients were exposed to harm.

**CONCLUSION:**

The detrimental impact of the COVID-19 pandemic on cancer care is widespread, with varying magnitude among centers worldwide. Additional research to assess this impact at the patient level is required.

## INTRODUCTION

Cancer is a serious disease that affects the lives of millions around the globe.^1^ Because of the nature of the disease and its treatment, patients with cancer are required to visit health care facilities more than patients with other diseases. The treatment of patients with cancer requires a full involvement of multidisciplinary teams throughout the disease trajectory from diagnosis to survivorship or end-of-life care.^2^ During the disease course, patients require multiple hospital visits for assessment by different clinicians and to undergo many laboratory or imaging tests for diagnosis, staging, or monitoring of treatment effects in addition to different types of procedures and interventions. Besides medical providers, patients with cancer need the help of many other disciplines, such as social workers, psychologists, educators, and other support services. Once diagnosed with cancer, patients need continued monitoring and support during and after treatment. To achieve the maximum benefits for patients, these services should be working in harmony and a timely fashion, with great commitment and compliance from the patients because any unjustifiable deviation from the well-established standards may lead to fragmented and poor-quality care and worse patient outcome.

**CONTEXT**

**Key Objective**
What was the impact of COVID-19 pandemic on cancer care at a global level? How did the magnitude of impact vary in different settings?
**Knowledge Generated**
Our study revealed that the overwhelming majority (88%) of the 356 participating centers on six continents faced challenges in providing usual cancer care for many reasons, including precautionary measures, an overwhelmed health care system, lack of personal protective equipment, and staff shortage. More than a third of these centers reported patient exposure to harm from interruption of cancer-specific care or other medical care. As expected, the impact was more pronounced in low-income countries. The implementation of virtual communication and remote care were prevalent responses in most centers.
**Relevance**
The lessons learned from this study may help oncology centers to manage the current pandemic more efficiently and be better prepared for any future crises.

The new coronavirus started as an outbreak in late 2019, and in a few weeks, it became a global pandemic.^3-5^ This new virus has a high contiguity rate and a fatality rate between 2% and 3%.^6-8^

Up to June 8, 2020, > 7,000,000 people infected and > 400,000 deaths were reported on a global scale, with large discrepancies in incidence and fatality among countries as reported by the COVID-19 Dashboard of the Center for Systems Science and Engineering at Johns Hopkins University.^9^

The COVID-19 pandemic affected health care services in many dimensions, starting from interrupting regular patient flow to health care facilities, stressing and overwhelming the health care resources, and leading to the implementation of extra protective measures and social distancing with increased utilization of telehealth and virtual medicine. As a precautionary approach, oncology practices implemented specific measures such as reducing the number of patients in outpatient clinics, reducing unnecessary or elective procedures, and discharging patients from inpatient services.^10,11^

Patients with cancer are a vulnerable population, and they are prone to many harms during such pandemics, including susceptibility to life-threatening infections and interruption of their cancer or usual medical care. Hence, oncologists have faced a major challenge to balance the delivery of high-quality continuous unfragmented cancer care with minimizing patients’ risk of exposure during care. The negative impact of the pandemic is likely to be greater in low and middle income countries with limited resources, poor infrastructure, shortage of health care providers and organized care teams, scarcity of medical supplies and personal protective equipment (PPE), and poor access to technology^12-14^—resulting in a lack of ability to provide and deliver critical care.

The responses of oncology centers to the pandemic and interventions implemented were reported on a limited scale by different centers, and reporting was done in general terms. To our knowledge, there is no systematic study that assessed these responses and the impact of the COVID-19 pandemic on cancer care. Our study aimed to evaluate the response of oncology centers and services to the pandemic at a global level and to assess the impact on cancer care delivery and implemented interventions.

## METHODS

### Study Design and Participants

This cross-sectional study was conducted by a consortium of researchers from different countries aiming to measure the patterns of cancer care during the COVID-19 pandemic and quantify the impact of the pandemic on various components of cancer care delivery. The survey included questions to assess the performance of oncology centers in different countries in response to the COVID-19 pandemic. The tool evaluated cancer services management during the crisis and the perception of oncologists about the potential harm to patients.

The survey was disseminated electronically using the SurveyMonkey platform (SurveyMonkey, San Mateo, CA) and targeted a convenience sample of individuals identified as key informants in their institutions who were aware of the management and updates about oncology practices and services during the pandemic. SurveyMonkey was chosen by the research team for its advanced design capabilities.

The key informants were senior oncologists who were active in the global oncology community and had a good network of contacts. We targeted oncologists who were involved in clinical care and aware of their center information. They were selected from different geographical regions and continents and acted as regional coordinators. The regional coordinators invited country coordinators to disseminate the survey to the targeted participants in their respective areas. Patients were not involved in this study.

### Procedures and Study Instrument

The data collection instrument (online survey) consisted of 51 English-language questions developed by the research team, which was composed of oncologists and research methodologists who have monitored cancer care and tracked key cancer care indicators across countries during the COVID-19 pandemic. Content validity of the instrument was assessed by presenting it to a group of eight experts (six oncologists, an epidemiologist, and a nurse manager). The group evaluated the relevance and appropriateness of each item of the scale, and a content validity ratio (0.79) and content validity index (0.87) were generated. All changes were corrected as suggested by the panel of experts. The obtained final version of the tool was further piloted on a sample of physicians who worked with patients with cancer in different institutions (n = 20) to ensure that all the items used were clear and understandable.

The online survey included characteristics of the surveyed centers (location of the center, type of services offered, and number of new patients served annually), interruption of cancer care (reasons for interrupting usual care and reducing or discontinuing services and access to products and medications), potential harm to patients as a result of interrupting services (missing treatment, cancellation of clinic visits, seeking care elsewhere, and type of harm that may have affected the patients), diagnosis and management of COVID-19 (diagnosed cases in the city, diagnosed patients without cancer, diagnosed patients with cancer, diagnosed cancer center staff, availability of PPE, and availability of practice guidelines for COVID-19 diagnosis and management), and virtual management of patients with cancer and remote care (availability of tumor boards, availability and management of virtual tumor boards, availability and management of virtual clinics, possibility of persistence of these services after the pandemic subsides, and dissemination of medication to patients with cancer).

The questions for this study were adapted from a literature review of the topic being measured, and respondents familiar with the concept of cancer care management were interviewed to help to generate instrument items. Content validity of the constructed instrument was ensured by the judgment of experts on the relevancy and clarity of the items. SurveyMonkey was used to disseminate the survey and compile the responses.

### Data Analysis

Responses were collected anonymously and recorded. Upon completion of the data collection process, data were imported from SurveyMonkey into Stata 14.0 software (StataCorp, College Station, TX) for statistical analysis. Descriptive analyses to examine the characteristics of the sample were performed. Means, standard deviations, and frequencies with their corresponding 95% CIs were reported for every surveyed cancer institution.

Ethical approval was obtained from the institutional review board at King Abdullah International Medical Research Center at the King Saud bin Abdulaziz University for Health Sciences. Comparative analysis was conducted to compare the severity of the pandemic impact on the centers based on the World Bank income classification of the responding countries, such as interruption of care, access to PPE and medications, use of virtual technology to run clinic visits or tumor boards, and delivery of medications to patients’ homes.

## RESULTS

**FIG 1:**
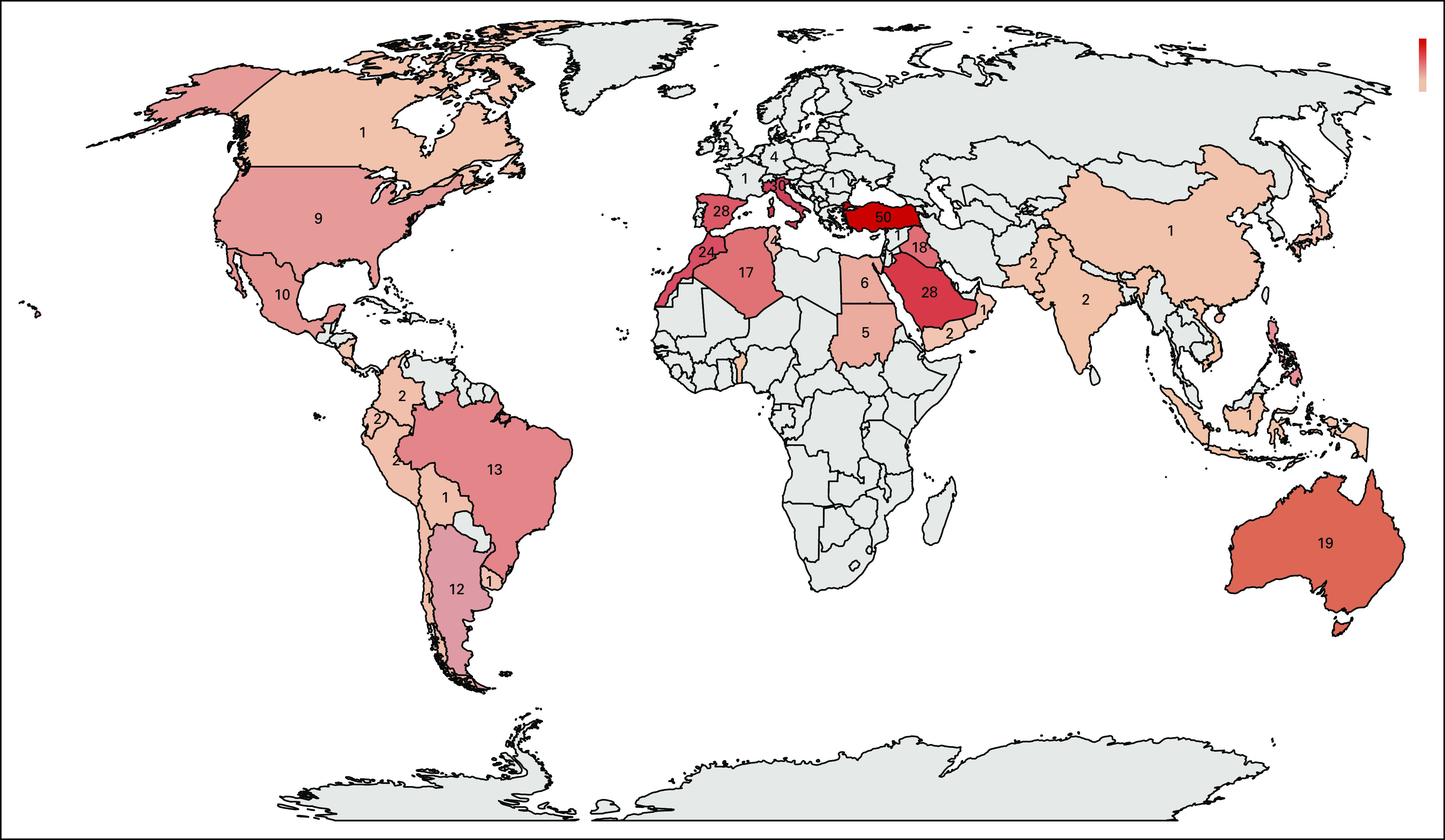
Number of the participating centers per country.

Between April 21 and May 8, 2020, responses were obtained from 356 centers from 54 countries on six continents: Africa, Asia, Australia, Europe, North America, and South America (Fig 1). Table 1 lists the characteristics of the participating centers, which shows representation of various countries, type of cancer services, and capacity. The reported number of new patients with cancer served in the participating centers was 716,979 per year.

**TABLE 1:**
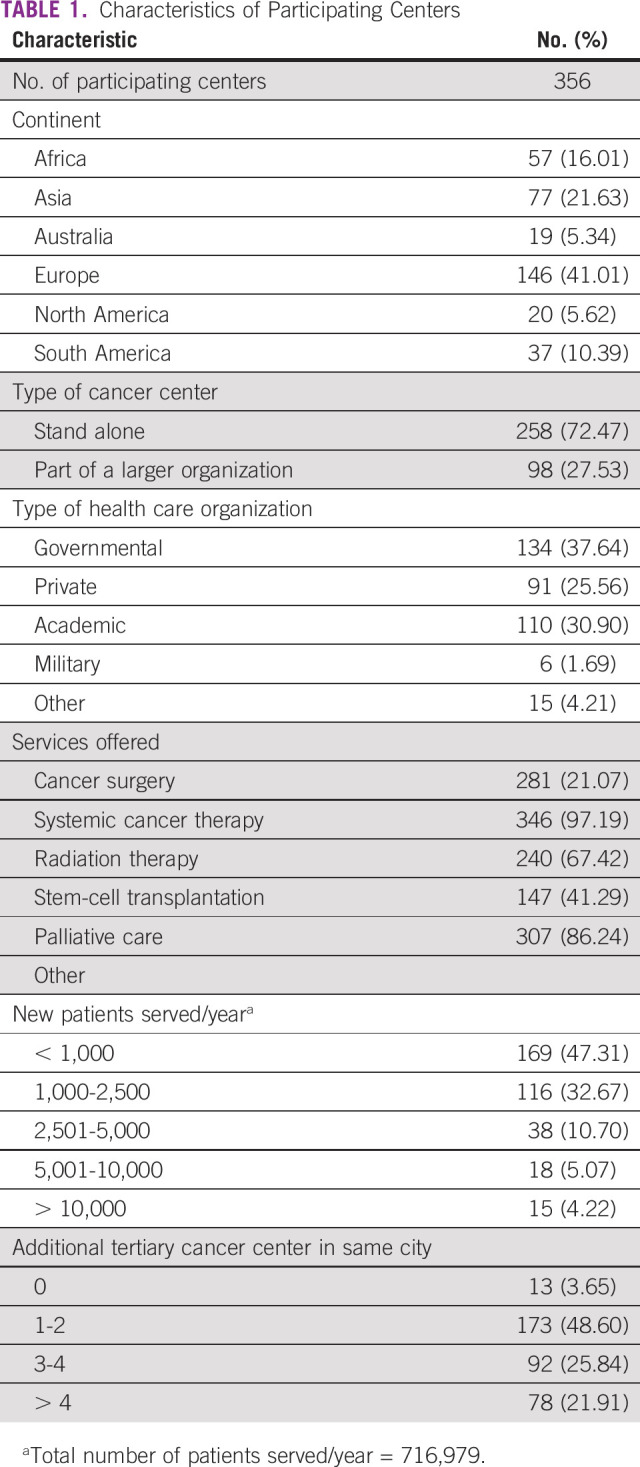
Characteristics of Participating Centers

Variable extent of service disruption was reported from some centers remaining fully functional as usual to others being completely closed. The majority of the centers (88.2%) reduced their usual level of care, and more than half (55.34%) of the reduction was a precautionary measure; however, in many cases, the disruption was due to other causes, such as an overwhelmed system (19.94%), staff shortage (17.98%), and lack of access to medications (9.83%; Table 2).

**TABLE 2:**
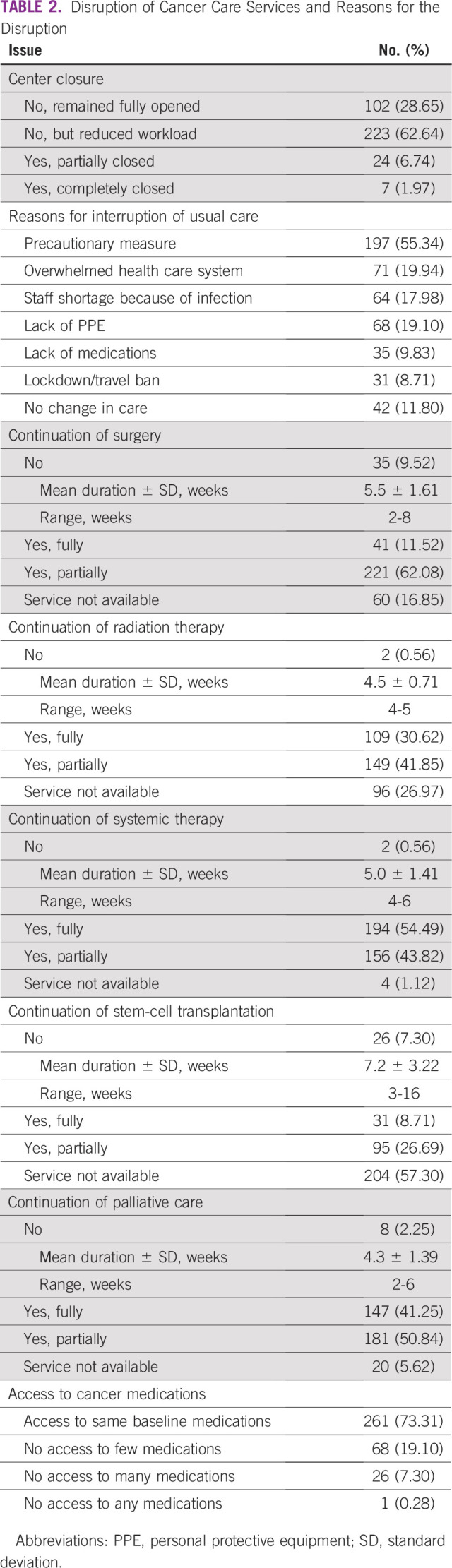
Disruption of Cancer Care Services and Reasons for the Disruption

Many patients missed chemotherapy sessions, with 46.35% of the centers reporting that more than 10% of their patients missed at least one session. As expected, many centers reduced their outpatient visits and switched to virtual clinics. Patients in many centers (58.15%) did not have the option of seeking care outside their centers. Participants reported the exposure of a significant fraction of patients to harm either from interruption of cancer-specific care or from interruption of care for other diseases (36.52% and 39.05%, respectively; Table 3). The reported harm estimates ranged from < 1% up to 80% of patients.

**TABLE 3:**
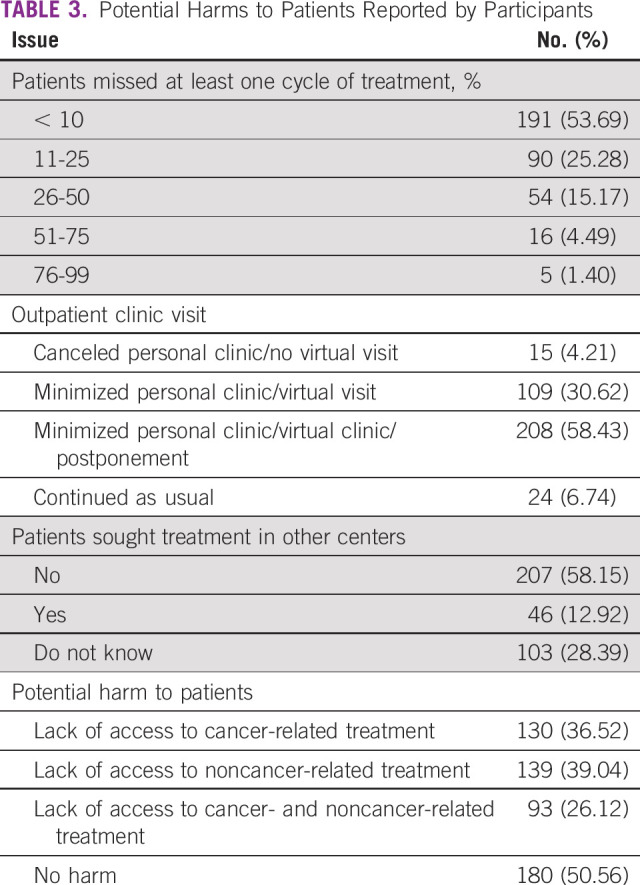
Potential Harms to Patients Reported by Participants

Cases of a confirmed COVID-19 diagnosis among patients were reported in 53.93% of the centers and among staff in 44.38% of the centers. Shortage of PPE was reported in multiple centers (48.31%). Physicians followed different guidelines for patient management and prioritization during the pandemic (Table 4).

**TABLE 4:**
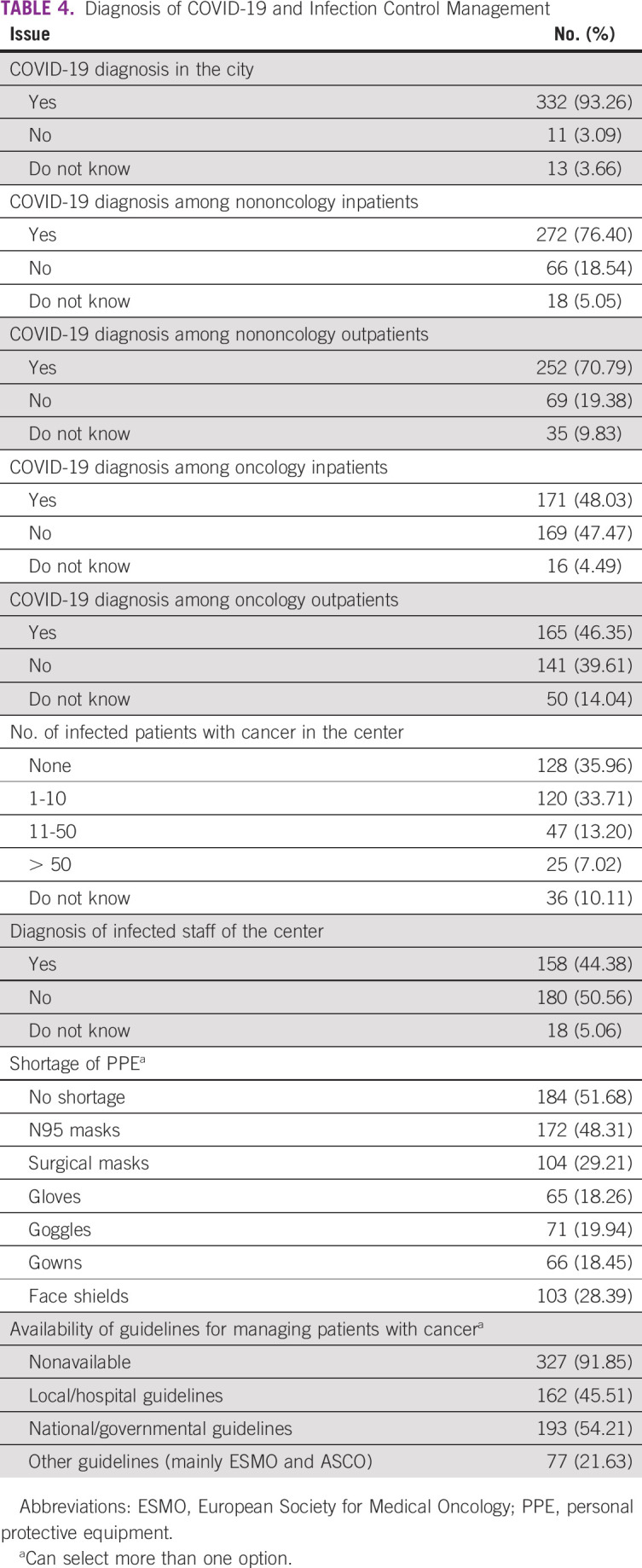
Diagnosis of COVID-19 and Infection Control Management

Most centers implemented virtual clinics and virtual tumor boards (77.53% and 84.27%, respectively), and many believed that these changes will remain active beyond the pandemic. Remote care included performing routine laboratory tests close to patients’ homes and shipping medications to them (Table 5). At the time of completing the survey, only 16% of the centers reported that work is back to prepandemic baseline.

**TABLE 5:**
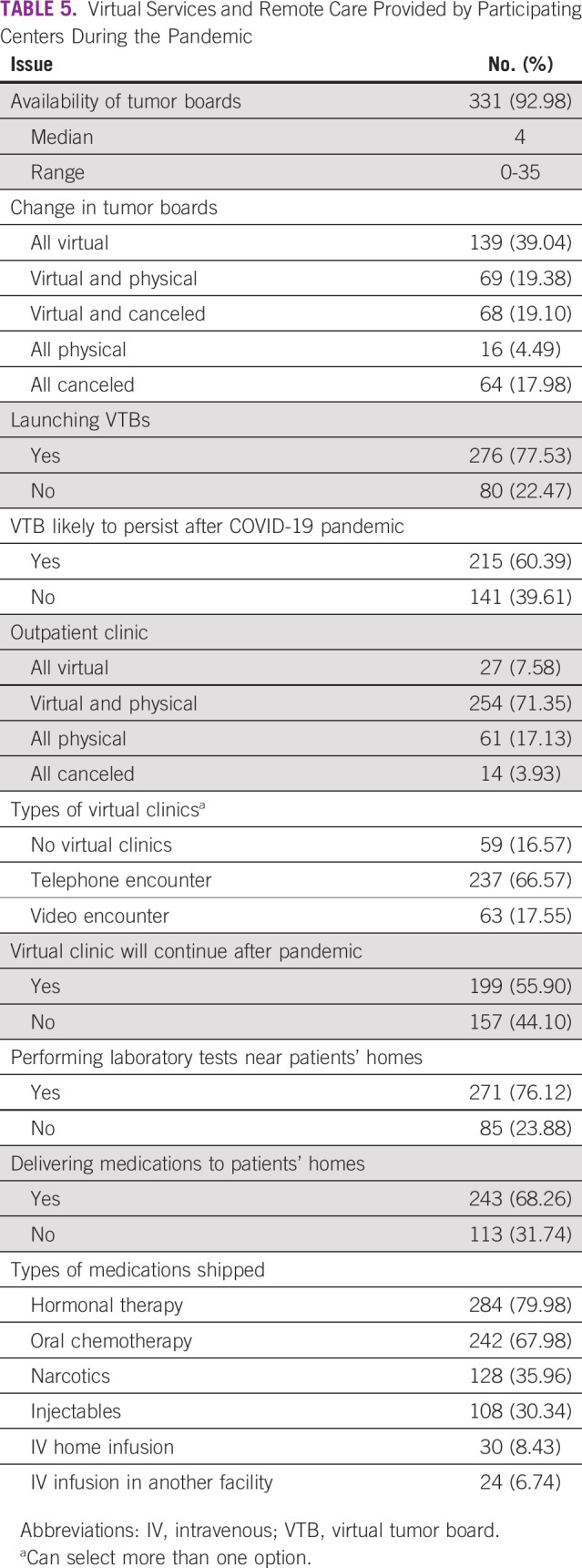
Virtual Services and Remote Care Provided by Participating Centers During the Pandemic

The severity of the pandemic impact on different aspects of care varied on the basis of the country level of income per World Bank stratifications, revealing a worse impact in centers of lower-resource countries. Lack of PPE, access to medications, and estimated exposure to harm was worse in lower-income countries. Furthermore, the centers in these countries were less likely to hold virtual tumor boards, run virtual clinics, do laboratory tests near patients’ homes, or deliver medications to patients (Table 6).

**TABLE 6:**
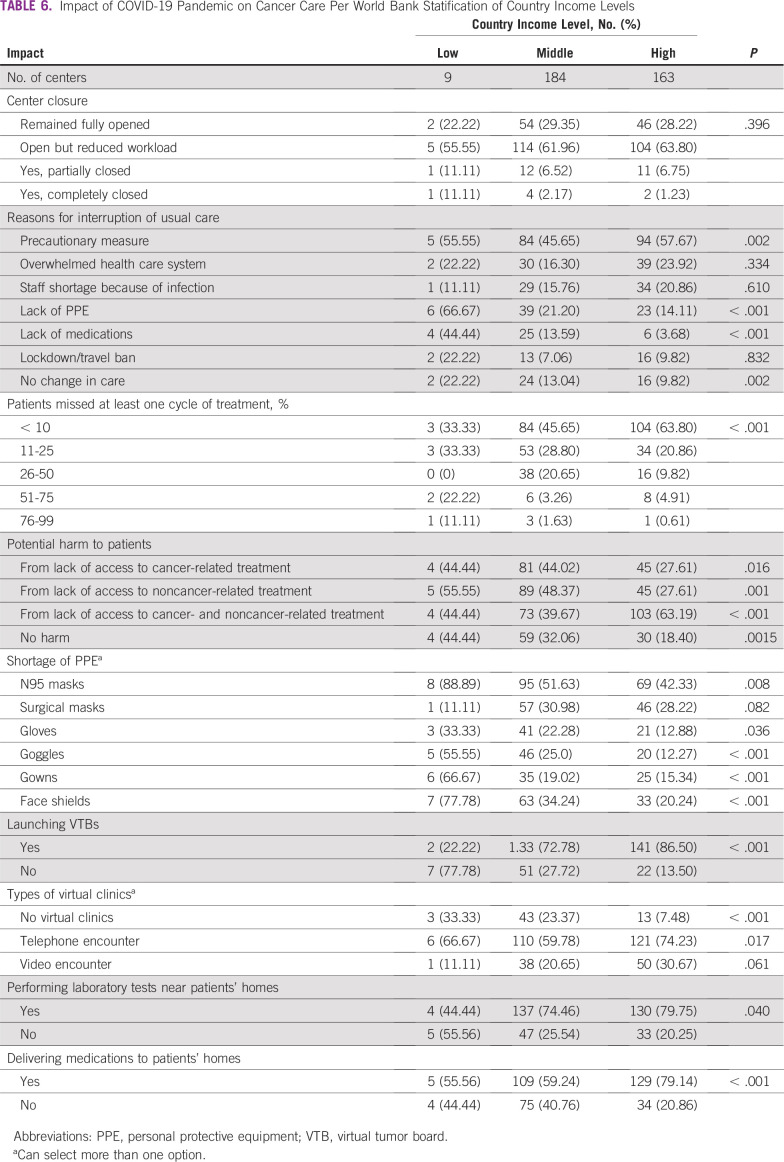
Impact of COVID-19 Pandemic on Cancer Care Per World Bank Statification of Country Income Levels

## DISCUSSION

Our study demonstrated the far-reaching impact of the COVID-19 pandemic on cancer services worldwide. Most of the centers faced challenges in maintaining the same level of care as before the pandemic, and therefore, they reduced or adjusted their services to different degrees using various approaches.

The main reason to reduce services was a precautionary measure to minimize patient visits and maintain social distancing. These are prudent actions recommended by many of the published guidelines and recommendations on managing patients with cancer during this pandemic.^15-19^ Many of these guidelines were made to the best judgment of authors and based on anecdotal experience as well as reports from frontline oncologists in the current pandemic or from those who had previous experience in infectious outbreaks such as Middle East respiratory syndrome and severe acute respiratory syndrome.^20^ However, because there were many unknown facts about the pandemic and how it would affect an individual country or even an individual institution—in addition to the inherent heterogeneity of patients with cancer and health care systems—many arbitrary decisions were made, and the impact of these decisions on patients care and outcome deserves further investigation to help to create an evidence-based approach for the future. On the other hand, there were other involuntary causes for reducing the level of care provided that are worth reflecting on to avoid them or at least minimize their impact during any future crisis, including staff shortage, PPE shortage, and lack of access to medications.

As frontline fighters of the pandemic, it is critical to manage health care staff well during the crisis and to be able to deliver care to all patients and prevent patients from exposure to different harms, such as infection, emotional disorders, and burnout.^21,22^ Shortage of PPE is a major concern because it exposes patients and health care staff to risk of infection or treatment interruption, compromises patient care, and leads to stress and discontent among staff.^23^ Addressing this issue requires a multilayered approach from all stakeholders, including the country’s government.^24-27^

Managing medication formulary during a crisis is an essential function of organization leaders to ensure continuity of delivery of timely treatment to patients with cancer. Pharmacy management should ensure that an adequate supply for cancer and noncancer medications is maintained.^28^ Major regulatory agencies, such as the US Food and Drug Administration and European Medicines Agency as well as the United Nations, have initiatives and guides to address drug shortages during a pandemic.^28-31^

With more than a third of the participants reporting potential harms to patients with cancer from the disruption of usual care, some centers reported that up to 80% of their patients had exposure to potential harms. Although these numbers varied among centers, patient harm was certainly encountered by many oncologists because of the pandemic. The exact magnitude should be determined with time and future systematic studies because there are different risks of harm, including issues related to cancer management and noncancer-related management of other medical conditions that affect patients with cancer. The spectrum of cancer-related harm is wide and includes halting screen-ing and prevention efforts, delaying timely diagnosis and staging of new patients, delaying initiation of therapy, interrupting ongoing treatment, delivering suboptimal palliative care, and disrupting clinical research.^32,33^

Limitations of the study include capturing the information in the midst of a pandemic, with variation in its severity in these countries and the full picture of pandemic impact still unclear. However, this study is important to paint the status of cancer care at a global level and will serve as a baseline for follow-up to assess the long-term effect of the pandemic on cancer care and outcome.

The study was completed by experts from these centers who provided their best estimates of certain data, such as patient harm, but this information is not backed by actual data, which are needed in future studies to get a better measurement of the real harm. In addition, participation in this study was voluntary and may have been skewed because responding physicians are willing to share information while nonresponding physicians may not be willing or able to do so for various reasons. The study may not have adequate representation from certain regions in the world such as sub-Saharan Africa and other regions, but the sample size helped us to perform an analysis that enabled us to draw plausible conclusions about the challenges encountered in poor countries with limited resources, emphasizing previous reports by others.^12,14,34^ Of note, scarcity of resources during the peak of the pandemic was encountered even in affluent countries with overwhelmed health care systems, which raises the need for a structured approach to resource management during such crises.^24,27,35,36^

As the pandemic evolves, we are gaining new knowledge and adjusting some older approaches, and this is valuable for the oncology field and health care systems in general.^37^ What we are sure of is that a new normal of health care, including oncology, will emerge after the pandemic. This new normal will involve more remote care; care closer to home; and more use of technology in care delivery, research, education, and business management. In addition, we may find that omitting cycles in maintenance therapy or fewer patient office visits or surveillance tests may not have a negative impact on outcome, although this will need prospective evaluation with properly conducted clinical studies.

Telehealth and digital health in oncology can be an excellent tool for real-time video consultations for primary care triage and interventions, such as counseling, medication prescribing and management, management of long-term treatment, and postdischarge coordination supported by remote monitoring capabilities. It can be also a useful tool for wellness interventions and in areas such as health education, physical activity, diet monitoring, health risk assessment, medication adherence, and cognitive fitness.^38^

Lessons learned from this pandemic should be become an integral part of the new normal of health care. The integration of cancer care as a part of the institutional emergency preparedness plan will improve patient outcomes in similar crises. The cancer care continuum should have a major component of effectively managing patients during pandemics or major crises. Thus, we not only avoid harms in any future pandemic but also use the momentum gained from the current one to improve overall health care delivery for our patients and enhance the quality of care across borders by large-scale collaborations among cancer care stakeholders.^38^ These collaborations and initiatives should aim to close the global gap in cancer care created by the disparity in access to resources and exacerbated by the pandemic. This can be achieved by a multipronged approach, including the use of technology and other innovative approaches to improve care not just across borders but even within the same country.^39-42^
